# In Situ-Grown Al_2_O_3_ Nanoflowers and Hydrophobic Modification Enable Superhydrophobic SiC Ceramic Membranes for Membrane Distillation

**DOI:** 10.3390/membranes14050117

**Published:** 2024-05-19

**Authors:** Yuqi Song, Kai Miao, Jinxin Liu, Yutang Kang, Dong Zou, Zhaoxiang Zhong

**Affiliations:** 1 School of Environmental Science and Engineering, Nanjing Tech University, Nanjing 211816, China; syq2023@njtech.edu.cn (Y.S.); mk@njtech.edu.cn (K.M.); jinxinliu@njtech.edu.cn (J.L.); kangyt@njtech.edu.cn (Y.K.); 2 National Engineering Research Center for Special Separation Membrane, College of Chemical Engineering, Nanjing Tech University, Nanjing 211816, China

**Keywords:** ceramic membrane, Al_2_O_3_ nanoflowers, superhydrophobic surface, vacuum membrane distillation

## Abstract

Membrane distillation (MD) is considered a promising technology for desalination. In the MD process, membrane pores are easily contaminated and wetted, which will degrade the permeate flux and salt rejection of the membrane. In this work, SiC ceramic membranes were used as the supports, and an Al_2_O_3_ micro-nano structure was constructed on its surface. The surface energy of Al_2_O_3_@SiC micro-nano composite membranes was reduced by organosilane grafting modification. The effective deposition of Al_2_O_3_ nanoflowers on the membrane surface increased membrane roughness and enhanced the anti-fouling and anti-wetting properties of the membranes. Simultaneously, the presence of nanoflowers also regulated the pore structures and thus decreased the membrane pore size. In addition, the effects of Al_2_(SO_4_)_3_ concentration and sintering temperature on the surface morphology and performance of the membranes were investigated in detail. It was demonstrated that the water contact angle of the resulting membrane was 152.4°, which was higher than that of the pristine membrane (138.8°). In the treatment of saline water containing 35 g/L of NaCl, the permeate flux was about 11.1 kg⋅m^−2^⋅h^−1^ and the salt rejection was above 99.9%. Note that the pristine ceramic membrane cannot be employed for MD due to its larger membrane pore size. This work provides a new method for preparing superhydrophobic ceramic membranes for MD.

## 1. Introduction

The problem of water scarcity is becoming severer due to industrial pollution and climate change. Desalination technology has the potential to alleviate the shortage of freshwater resources. Membrane distillation (MD) technology, as an emerging desalination technique, is receiving increasing attention because of its high separation efficiency and mild operating conditions [1,2,3]. MD technology is a separation technique that uses hydrophobic porous membranes to separate saline water. These membranes have a microporous structure. In the MD process, one side of the membrane is the feed side that directly contacts with the feed solution, while the other side is the permeate side. Under the action of vapor pressure difference, the membrane can selectively allow water vapor to pass through and block the passage of saline water, thereby realizing the separation of water and salt [4,5,6,7]. Therefore, the salt rejection can theoretically reach 100%.

The commonly used membranes are generally polymer membranes, such as polyvinylidene fluoride (PVDF), polypropylene (PP), polyethylene (PE) and polytetrafluoroethylene (PTFE) membranes [8,9,10]. However, polymer membranes cannot be employed under critical conditions for a long time due to their poor chemical and mechanical properties. In contrast, ceramic membranes have better thermal stability and mechanical stability, and can also be employed under harsh conditions [11,12,13]. As a result, ceramic membranes have gradually attracted attention in MD applications [14]. However, ceramic membranes are hydrophilic due to the existence of a large number of hydroxyl groups on the surface, so it is necessary to modify the surface of ceramic membranes to make them hydrophobic [15]. The commonly used method at present is to directly modify the membrane hydrophobicity using fluorosilane. However, membranes modified directly through traditional methods often exhibit poor hydrophobicity and are prone to being wetted. Wei et al. used a conventional hydrophobic modification method to modify the ceramic membrane and found that asymmetric porous cordierite ceramic membranes directly modified by 1H,1H,2H,2H-perfluorodecyl -triethoxysilane (PFDTS) experienced severe wetting after a 6 h MD process [16]. How to improve the stability and hydrophobicity of ceramic membranes in the MD process is challenging.

As is well known to all, surface roughness plays a crucial role in the hydrophobicity of membranes [17]. Based on the Cassie theory, rough micro-nano structures can trap more air, forming an air layer on the membrane surface, thereby reducing the contact area between the liquid and the membrane surface, making it easy for the liquid to slip off the membrane surface [18,19,20]. Currently, enhancing membrane roughness is mainly achieved by depositing inorganic nano-sized particles (such as ZnO, TiO_2_, SiO_2_, CuO, etc.) on the membrane surface to construct a reentrant structure [21,22,23]. For example, Chen et al. [24] deposited ZnO nanorods and nanoparticles on the surface of Al_2_O_3_ hollow fiber membranes using the chemical bath deposition method, increasing the roughness from 93.41 nm to 186.34 nm, enhancing the wetting resistance of the membrane. Mohd et al. [25] synthesized TiO_2_ nanoflowers and nanorods on mullite hollow fiber membranes via the hydrothermal method, increasing the roughness from 51.4 nm to 136.8 nm. The prepared membrane obtained a satisfactory water contact angle of 162°, demonstrating excellent hydrophobic properties. However, there is currently limited research on the application of the in situ growth of Al_2_O_3_ nanoflowers on ceramic membranes via the hydrothermal method for MD.

**Figure 1:**
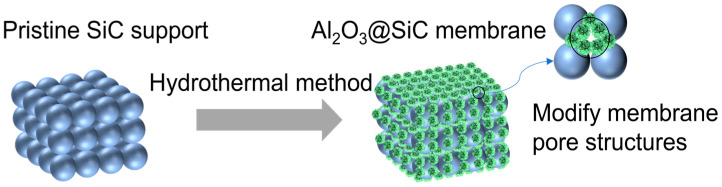
Scheme of the in situ growth of Al_2_O_3_ nanoflowers.

In this work, the hydrothermal method was utilized successfully to obtain the in situ growth of Al_2_O_3_ nanoflowers with different scales on SiC membranes. Additionally, the membranes were modified using organosilane to lower the surface energy to fabricate superhydrophobic Al_2_O_3_@SiC composite ceramic membranes. These nanoflowers cannot only increase the membrane roughness but also regulate the pore structures of the membrane (see Figure 1). The concentration of the Al^3+^ precursor and the sintering temperature were investigated in detail for their impact on the microstructure of the resulting membrane. The prepared composite ceramic membranes were also characterized using SEM, XRD, XPS, etc. Moreover, the vacuum membrane distillation (VMD) performance of the prepared membranes was investigated.

## 2. Materials and Methods

### 2.1. Materials

Aluminum sulfate (Al_2_(SO_4_)_3_, AR) was purchased from Meryer Chemical Technology Co., Ltd. (Shanghai, China). Urea (CO(NH_2_)_2_, 99%); 1H,1H,2H,2H-perfluorodecyl -triethoxysilane (PFDTS, 98%) and sodium chloride (NaCl, 99.5%) were purchased from Shanghai Aladdin Biochemical Technology Co., Ltd. (Shanghai, China). SiC supports were fabricated from SiC powders with a particle size of 5 microns. Deionized (DI) water produced by a pure water machine of Nanjing Yuheng Instrument and Equipment Co., Ltd (Nanjing, China) was used in all experiments.

### 2.2. Preparation of Superhydrophobic SiC Ceramic Membranes

#### 2.2.1. Preparation of SiC Supports

SiC supports were fabricated from SiC powders with a particle size of 5 microns. Five-micron SiC powders were used as the supporting aggregates, and an 8 wt% PVA solution was used as binders. After thorough mixing, the green body (unsintered SiC support) was obtained by a dry pressing process with the pressing time of 40 s and holding pressure of 10 MPa. Subsequently, SiC supports were obtained through sintering at 1300 °C. SiC supports are circular in shape, and they are discs (the thickness of the disc is 2.1 mm and the diameter is 31 mm).

#### 2.2.2. In Situ Growth of Al_2_O_3_ Nanoflowers

The growth of Al_2_O_3_ nanoflowers on the surface of SiC membranes was first carried out using the hydrothermal method, followed by sintering. The pristine SiC supports were washed by ethanol for 5 min and dried at 60 °C for 30 min. The precursor solution was prepared by adding Al_2_(SO_4_)_3_ (with concentrations of 0.0875 M, 0.175 M, 0.2625 M) and urea (with the concentration of 2.22 M) into 30 mL of deionized (DI) water. Then, the solution was stirred thoroughly to obtain a clear and transparent solution. The SiC supports were vertically placed into the Teflon lining and then the precursor solution was poured into it. Subsequently, the SiC supports were reacted at 160 °C for 8 h. Then, the membranes were washed thoroughly by DI water. Subsequently, membranes were dried under a vacuum for 24 h. After that, membranes were sintered at different temperatures (600 °C, 900 °C or 1200 °C) for 12 h.

#### 2.2.3. Hydrophobic Modification

Firstly, a 2 vol% C8 solution was prepared by adding 1 mL of 1H,1H,2H,2H-Perfluorodecyl-triethoxysilane into 49 mL of ethanol. Secondly, the fluorination solution was sonicated by a sonicator for 5 min to ensure uniform mixing. Then, the prepared SiC ceramic membranes were immersed in the fluorination solution at 60 °C for 48 h. Subsequently, membranes underwent condensation polymerization at 120 °C for 3 h. The specific sample codes with different reaction processes are detailed in Table 1.

### 2.3. Characterization

Membrane surface morphology was examined by desktop scanning electron microscopy (Hitachi TM3000, Hitachi, Japan) and field-emission SEM (Hitachi S-4800, Hitachi, Japan). Pore size distribution of the membranes was measured using a capillary flow porometer (PMI ipore 1500, PMI, Ithaca, NY, USA). The water contact angle was measured by Dataphysios OCA25 (OCA 25, Dataphysios, Filderstadt, Germany). Surface roughness was observed by a surface profiler (PARK XE-100, Park Systems, Santa Clara, CA, USA). X-ray diffraction analysis was conducted by an X-ray diffractometer (RIGAKU MiniFlex600, Rigaku, Tokyo, Japan). The surface elements of the membranes were analyzed by X-ray Photoelectron Spectroscopy (XPS, Thermo Fisher Scientific Escalab, Waltham, MA, USA).

### 2.4. Vacuum Distillation Experiment

**Figure 2:**
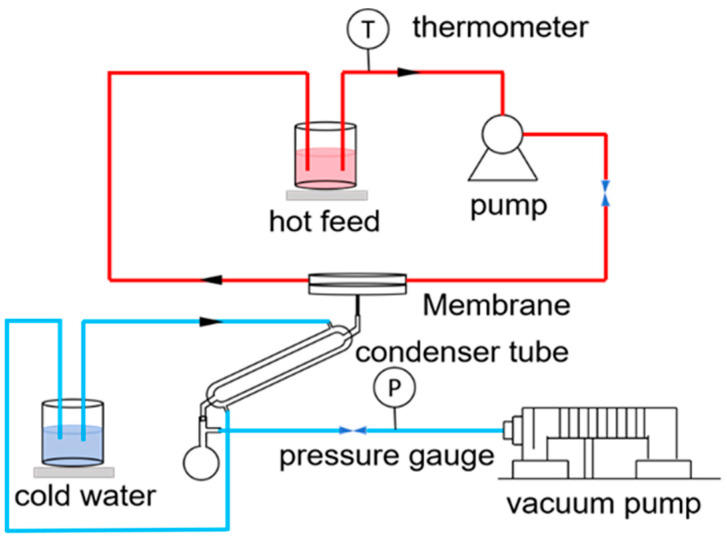
Schematic diagram of the VMD apparatus.

VMD tests were carried out using a laboratory-made setup. The membrane distillation setup is shown in Figure 2. In the MD application, the feed solution was heated in a water bath at 70 °C and then flowed under the action of a peristaltic pump. The flow rate was 200 mL/min. The vacuum degree was −0.090 MPa. When the feed liquid contacted the membrane surface, generated water vapor permeated through the membrane and condensed into liquid water. After a fixed period of time, the weight of the liquid in the flask was measured, along with its conductivity

Equation (1) can be used to calculate the permeate flux J (kg·m^−2^·h^−1^) of the membranes:

(1) $$J=\frac{m}{A\cdot ∆t}$$

where m represents the mass of the permeate water (kg), A represents the effective area of the membranes (m^2^) and Δt is the interval time (h).

Equation (2) can be used to calculate the salt rejection R (%) of the membranes:

(2) $$R=\frac{C_{1}-C_{2}}{C_{1}}\times 100\%$$

where R represents the salt rejection (%), C_1_ represents the conductivity of the feed solution (uS/cm) and C_2_ represents the conductivity of the resulting water.

## 3. Results and Discussion

### 3.1. Characterization of Pristine SiC Support

**Figure 3:**
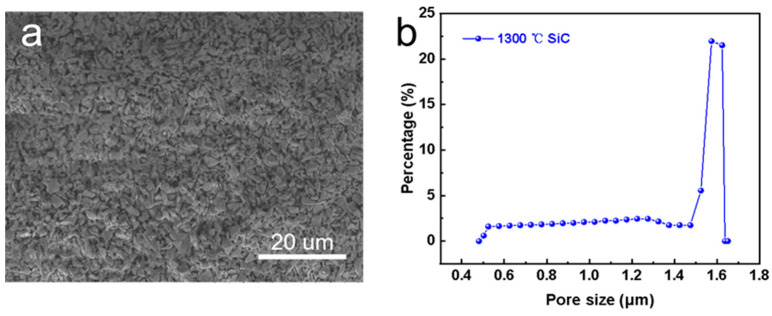
Characterization of pristine SiC support. (**a**) Surface morphology, and (**b**) pore size distribution.

Figure 3a displays the surface morphology of the pristine SiC support, which consisted of irregular particles. The membrane surface was smooth without any obvious defects. Figure 3b shows the pore size distribution of the SiC support. It can be seen that the average pore size was about 1.56 μm. Note that the pore size of the membrane was large (>1 μm) and the feed solution can easily wet the membrane [26]. Thus, the pristine SiC support was not suitable for MD application directly.

### 3.2. Effects of Al_2_(SO_4_)_3_ Concentration

**Figure 4:**
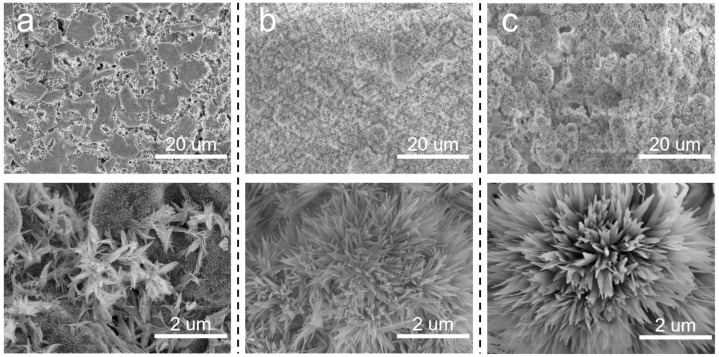
Surface morphologies of Al_2_O_3_@SiC composite membranes fabricated with different Al_2_(SO_4_)_3_ concentrations. (**a**) 0.0875 M, (**b**) 0.175 M and (**c**) 0.2625 M.

Based on the above discussion, it is meaningful to decrease the pore size of the SiC support. In situ growth of inorganic nanoflowers can regulate the pore size and surface roughness simultaneously. Herein, Al_2_O_3_ nanoflowers were proposed to decorate the membrane structures. The concentration of Al_2_(SO_4_)_3_ in the precursor solution significantly influenced the in situ growth of Al_2_O_3_ nanoflowers. When the sintering temperature was 600 °C, the surface morphologies of membranes prepared using varying Al_2_(SO_4_)_3_ concentrations are illustrated in Figure 4. At an Al_2_(SO_4_)_3_ concentration of 0.0875 M, the formation of a flower-like structure was not prominent. Instead, leaf-like structures were observed, which were inadequately distributed on the membrane surface and the surface remained relatively smooth. Additionally, there were still some macropores to be observed on the membrane surface. When the concentration of Al_2_(SO_4_)_3_ was 0.175 M, nanoflowers were evenly distributed on the membrane surface. When the Al_2_(SO_4_)_3_ concentration was increased to 0.2625 M, more nanoflowers appeared on the membrane surface, resulting in a hierarchical structure. As the Al_2_(SO_4_)_3_ concentration increased, there was a gradual increase in both the number and size of nanoflowers. This could be attributed to the low concentration of Al_2_(SO_4_)_3_, which failed to provide sufficient Al^3+^ to react effectively with urea. 

**Figure 5:**
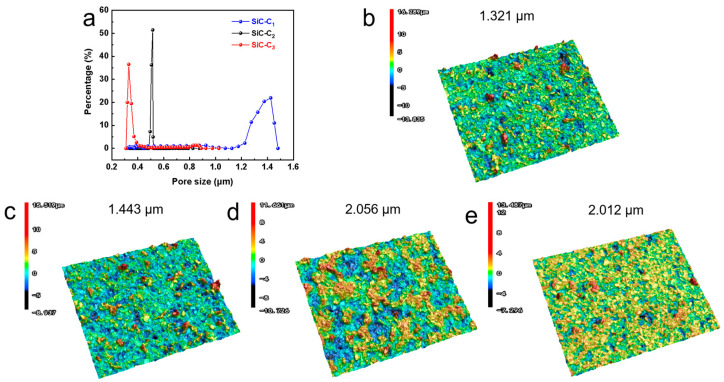
(**a**) Pore size distribution. Three-dimensional surface morphology of (**b**) pristine, (**c**) SiC-C_1_, (**d**) SiC-C_2_ and (**e**) SiC-C_3_.

To further determine the concentration of Al_2_(SO_4_)_3_, the pore size distributions of the membranes with different Al_2_(SO_4_)_3_ concentrations were investigated (see Figure 5a). The mean pore sizes of SiC-C_1_, SiC_2_ and SiC-C_3_ were around 1.37 um, 0.51 um and 0.37 um, respectively. The grown nanoflowers modified the membrane pore structures and thus decreased the membrane pore size, which was consistent with the SEM image in Figure 4. The surface roughness plays an important role in the hydrophobic properties of the membranes. The surface roughnesses of the prepared membranes were characterized by the surface profiler (See Figure 5b–e). The surface roughness of the pristine support was only 1.321 μm. After the in situ growth of Al_2_O_3_ nanoflowers on the membrane surface, the surface roughness of the membrane initially increased and then remained stable. The roughness of the SiC-C_1_ was only 1.443 µm. When the Al_2_(SO_4_)_3_ concentration was 0.175 M, the roughness increased to 2.056 um. When the concentration of Al_2_(SO_4_)_3_ further increased to 0.2625 M, the membrane roughness did not have an obvious change, with a roughness of 2.012 μm. The membrane roughness increased after the in situ growth of Al_2_O_3_ nanoflowers compared to the pristine supports. A rough membrane surface can store more air, leading to the presence of air pockets between the membrane surface and the feed liquid, and then reduce the contact area, enhancing the hydrophobic performance of the membranes. In summary, the most suitable concentration of Al_2_(SO_4_)_3_ was 0.175 M.

### 3.3. Effects of Sintering Temperature

**Figure 6:**
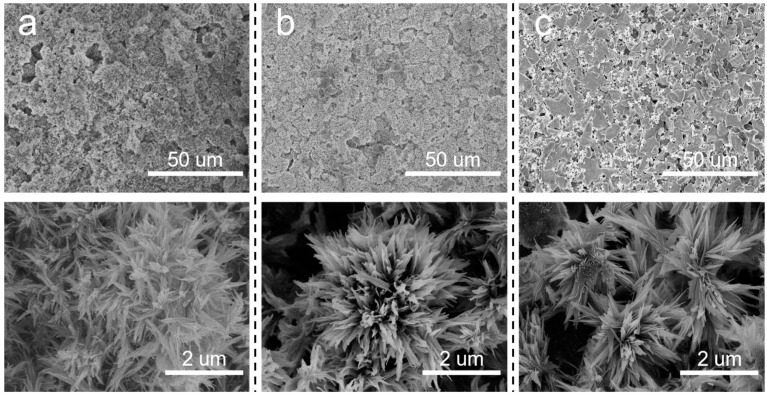
Surface morphology of Al_2_O_3_@SiC composite membranes fabricated at different sintering temperatures. (**a**) 600 °C, (**b**) 900 °C and (**c**) 1200 °C.

**Figure 7:**
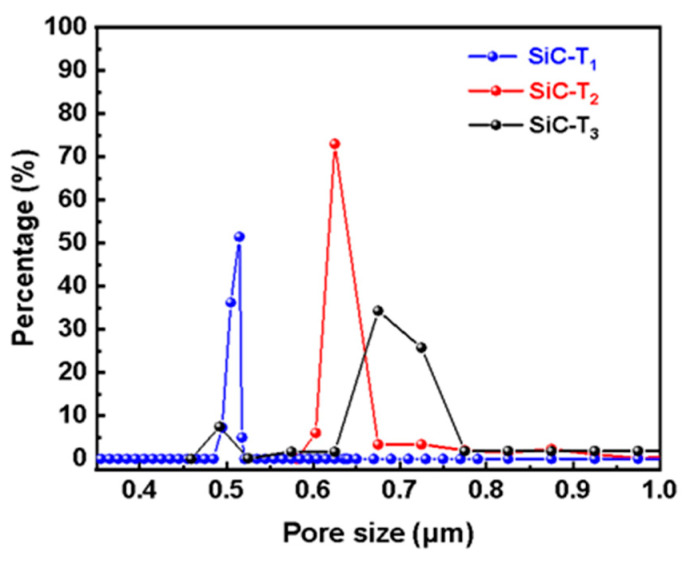
Pore size distribution of Al_2_O_3_@SiC composite ceramic membranes fabricated at different sintering temperatures.

In addition to the concentration of the precursor solution, the effects of the sintering temperature after the hydrothermal treatment on the prepared membranes were also investigated. It can be observed from Figure 6 that flower-like nanostructures were formed on the membrane surfaces at different sintering temperatures. However, as the sintering temperature rose, the number and size of flower-like structures on the membrane surface gradually decreased. SiC-T_1_ and SiC-T_2_ exhibited a significant presence of nanoflowers on the surface of membranes, while the surface of SiC-T_3_ appeared smoother with noticeable block-like structures of SiC particles, and the nanoflowers mainly existed within the pores formed by SiC particles. When the precursor concentrations were the same, the size of the formed nanoflower via the hydrothermal method remained the same. However, as the sintering temperature increased, the resulting crystal structure exhibited a larger apparent density, leading to a reduction in size and number after being sintered at higher temperatures. It can be observed from Figure 7 that the average pore sizes of SiC-T_1_, SiC-T_2_ and SiC-T_3_ were 0.51 um, 0.62 um and 0.71 um, respectively. As the sintering temperature rose, the average pore size of the Al_2_O_3_@SiC composite ceramic membranes increased continuously, which was also in line with the SEM images. In summary, the most suitable sintering temperature was 900 °C.

### 3.4. XRD Analysis of Al_2_O_3_ Nanoflowers Synthesized via Hydrothermal Method

**Figure 8:**
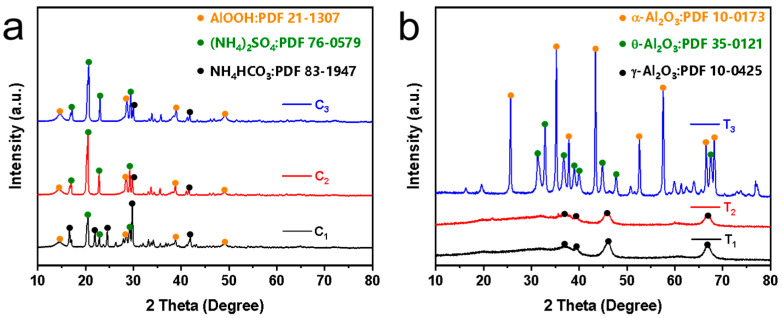
XRD patterns: (**a**) Unsintered powders synthesized by different Al_2_(SO_4_)_3_ concentrations, and (**b**) powders sintered at different temperatures. Note that C_1_, C_2_ and C_3_ represent Al_2_(SO_4_)_3_ concentrations of 0.0875 M, 0.175 M and 0.2625 M, respectively. T_1_, T_2_ and T_3_ represent powders sintered at 600 °C, 900 °C and 1200 °C, respectively.

The XRD patterns in Figure 8a show the unsintered Al_2_O_3_ nanoflower powders obtained from the hydrothermal reaction of urea and varying concentrations of Al_2_(SO_4_)_3_ at 160 °C for 8 h. It can be seen that the XRD patterns of the obtained powders are similar at different Al_2_(SO_4_)_3_ concentrations with diffraction characteristic peaks appearing at 14.48°, 28.18°, 38.34° and 48.93°, respectively. Compared with the diffraction card of boehmite (JCPDS 21-1307), it was found that they corresponded to the (020), (120), (031) and (200) crystal planes of the boehmite phase. This was attributed to the hydrolysis of urea in the autoclave at 160 °C that generated OH^−^ and NH_4_^+^. Subsequently, Al^3+^ reacted with OH^−^ to form Al(OH)_3_ colloidal. The Al(OH)_3_ colloid dissolved and recrystallized in the hydrothermal solution, ultimately forming boehmite microcrystals [27]. Note that the powders also contained byproducts such as ammonium sulfate and ammonium bicarbonate.

Figure 8b shows the XRD patterns of the powders sintered at different temperatures. It was observed that the XRD patterns of powders sintered at 600 °C and 900 °C were similar with diffraction characteristic peaks appearing at 37.60°, 39.49°, 45.86° and 67.03°, respectively. Compared with the diffraction standard card of γ-Al_2_O_3_ (JCPDS 10-0425), it was found that they corresponded to the (311), (222), (400) and (440) crystal planes of γ-Al_2_O_3_, respectively. When the powders were sintered at 1200 °C, the XRD pattern corresponded to the diffraction standard cards of θ-Al_2_O_3_ (JCPDS 35-0121) and α-Al_2_O_3_ (JCPDS 10-0173). The peaks observed at 31.47°, 32.78°, 36.68°, 38.92°, 39.86°, 44.83°, 47.62° and 67.42° corresponded to the (004), (20-2), (111), (104), (20-4), (21-1), (006) and (215) crystal planes of θ-Al_2_O_3_ (JCPDS 35-0121). Additionally, the peaks at 25.58°, 35.13°, 37.78°, 43.36°, 57.52°, 66.55°, 68.19° and 76.88° corresponded to the (012), (104), (110), (113), (116), (214) and (300) crystal planes of α-Al_2_O_3_ (JCPDS 10-0173) [28]. This indicated that the in situ-synthesized nanoflowers sintered at different temperatures were Al_2_O_3_. When the sintering temperature was 600 °C and 900 °C, γ-Al_2_O_3_ was formed on the membrane surface, and when the sintering temperature reached 1200 °C, α-Al_2_O_3_ and θ-Al_2_O_3_ were formed on the membrane surface.

### 3.5. Hydrophobic Modification and Membrane Distillation

**Figure 9:**
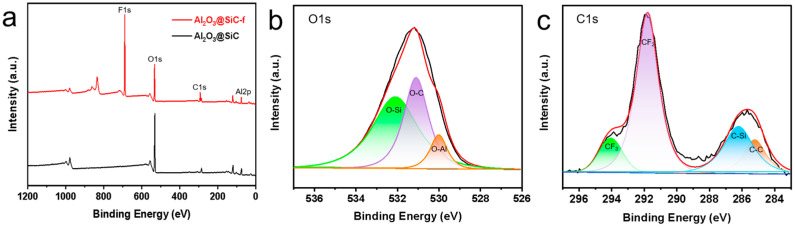
(**a**) XPS spectra of Al_2_O_3_@SiC membranes and Al_2_O_3_@SiC-f membranes, (**b**) decomposition of O1s in Al_2_O_3_@SiC membranes and (**c**) decomposition of C1s in Al_2_O_3_@SiC-f membranes.

After investigating the fabrication parameters of the membranes, the Al_2_O_3_ nanoflower-grown SiC composite membranes were produced successfully under the optimized conditions. Finally, the membranes were hydrophobically modified and characterized. XPS was carried out to analyze the chemical composition of the membrane surface deposited by Al_2_O_3_ nanoflowers. As shown in Figure 9a, two peaks were observed at 74.38 eV and 531.2 eV in the spectrum of the non-fluorinated membranes, corresponding to Al2p and O1s, respectively. Additionally, a peak was observed at around 689.09 eV in the spectrum of the fluorinated membranes (corresponding to F1s). The O1s spectrum of the non-fluorinated membranes is shown in Figure 9b with a prominent peak at 530.1 eV (assigned to O-Al), indicating the successful deposition of Al_2_O_3_ on the membrane surface [29]. Figure 9c displays the C1s spectrum of the fluorinated membranes, showing two prominent peaks at 294.78 eV (assigned to CF3) and 292.1 eV (assigned to CF2), indicating successful fluorination [30].

**Figure 10:**
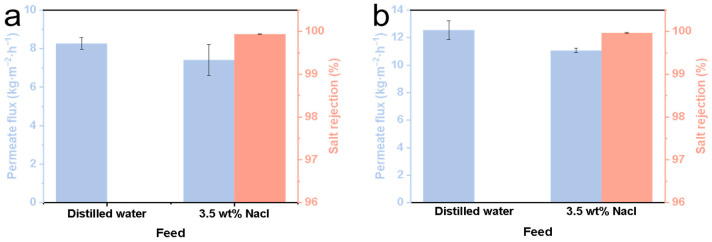
Permeate flux of (**a**) SiC-T_1_-f and (**b**) SiC-T_2_-f.

The SiC-T_1_-f, SiC-T_2_-f and SiC-T_3_-f membranes were selected for MD application. Note that the data reported in Figure 10 were obtained after a 12 h MD process.Due to the in situ growth of numerous micro-nanostructures on the surface, the contact angle of the SiC-T_1_-f membrane can reach 152.7° (see Table 2), but the initial permeate flux of the SiC-T_1_-f membrane was exceptionally low when treating saline water containing 35 g/L of NaCl at 70 °C (only 7.41 kg⋅m^−2^⋅h^−1^) (see Figure 10a). SiC-T_3_-f’s contact angle can reach 150.5° (see Table 2). Because of the presence of some large membrane pores, the feed can directly pass through SiC-T_3_’s pores, resulting in a lower salt rejection. By contrast, SiC-T_2_-f was more appropriate for MD. Aside from the appropriate pore size distribution and distribution density of Al_2_O_3_ nanoflowers, SiC-T_2_-f′s water contact angle also reached 152.4° (see Table 2). When the feed solution was distilled water, SiC-T_2_-f’s permeate flux was 12.5 kg⋅m^−2^⋅h^−1^. In the treatment of saline water containing 35 g/L of NaCl at 70 °C, the permeate flux was about 11.1 kg⋅m^−2^⋅h^−1^ and the salt rejection was above 99.9% (see Figure 10b). Table 3 displays the ceramic membrane properties of MD reported in previous works. It is demonstrated that the water flux of the resulting ceramic membranes in this work is satisfactory.

## 4. Conclusions

In this work, superhydrophobic Al_2_O_3_@SiC composite ceramic membranes were fabricated successfully. The presence of nanoflowers cannot only regulate the pore structures, but also increase the surface roughness, thus improving hydrophobicity and enhancing resistance to fouling and wetting. The effects of precursor solution concentration and sintering temperature on the morphology and performance of membranes were investigated. An appropriate preparation condition was determined through SEM, pore size distribution and MD performance. Results show that the optimal concentration of Al_2_(SO_4_)_3_ was 0.175 M and the optimal sintering temperature was 900 °C. The roughness of the prepared membrane increased from 2.056 μm to 1.321 μm and the water contact angle was about 152.4°. In the treatment of saline water containing 35 g/L of NaCl at 70 °C, the permeate flux was about 11.1 kg⋅m^−2^⋅h^−1^ and the salt rejection was above 99.9%. Currently, there are few reports on the growth of Al_2_O_3_ nanoflowers on ceramic membranes. This work provides a new approach for preparing superhydrophobic ceramic membranes. However, the long-term stability of this membrane is not satisfactory that should be further investigated in detail by finely-tuning the pore size of the ceramic support and microstructures of the membrane.

## Figures and Tables

**Table 1:** Sample codes with different reaction processes.

Sample Codes	Al_2_(SO_4_)_3_ Concentration (M)	Sintering Temperature (°C)	Fluorinated/ Unfluorinated
SiC-C_1_	0.0875	600	Unfluorinated
SiC-C_2_/SiC-T_1_	0.175	600	Unfluorinated
SiC-C_3_	0.2625	600	Unfluorinated
SiC-T_2_	0.175	900	Unfluorinated
SiC-T_3_	0.175	1200	Unfluorinated
SiC-C_1_-f	0.0875	600	Fluorinated
SiC-C_2_-f/SiC-T_1_-f	0.175	600	Fluorinated
SiC-C_3_-f	0.2625	600	Fluorinated
SiC-T_2_-f	0.175	900	Fluorinated
SiC-T_3_-f	0.175	1200	Fluorinated

**Table 2:** Contact angle of the membranes.

Sample Codes	Contact Angle (°)
SiC	138.8
SiC-T_1_-f	152.7
SiC-T_2_-f	152.4
SiC-T_3_-f	150.5

**Table 3:** Ceramic membrane properties for MD reported in previous works.

Refs.	Membrane Material	Concentration of the NaCl/g L^−1^	Permeate Flux/kg⋅m^−2^⋅h^−1^	Salt Rejection/%
[31]	Si_3_N_4_	20	12	99
[32]	SiC	50	0.13	98
[25]	Mullite	35	4.32	99.99
This work	SiC	35	11.1	99.9

## Data Availability

Data are contained within the article.
